# Land Use, Macroalgae, and a Tumor-Forming Disease in Marine Turtles

**DOI:** 10.1371/journal.pone.0012900

**Published:** 2010-09-29

**Authors:** Kyle S. Van Houtan, Stacy K. Hargrove, George H. Balazs

**Affiliations:** 1 Pacific Islands Fisheries Science Center, National Oceanic and Atmospheric Administration (NOAA) Fisheries Service, Honolulu, Hawaii, United States of America; 2 Nicholas School of the Environment and Earth Sciences, Duke University, Durham, North Carolina, United States of America; NIWA, New Zealand

## Abstract

Wildlife diseases are an increasing concern for endangered species conservation, but their occurrence, causes, and human influences are often unknown. We analyzed 3,939 records of stranded Hawaiian green sea turtles (*Chelonia mydas*) over 28 years to understand fibropapillomatosis, a tumor-forming disease linked to a herpesvirus. Turtle size is a consistent risk factor and size-standardized models revealed considerable spatial and temporal variability. The disease peaked in some areas in the 1990s, in some regions rates remained constant, and elsewhere rates increased. Land use, onshore of where the turtles feed, may play a role. Elevated disease rates were clustered in watersheds with high nitrogen-footprints; an index of natural and anthropogenic factors that affect coastal eutrophication. Further analysis shows strong epidemiological links between disease rates, nitrogen-footprints, and invasive macroalgae and points to foraging ecology. These turtles now forage on invasive macroalgae, which can dominate nutrient rich waters and sequester environmental N in the amino acid arginine. Arginine is known to regulate immune activity, promote herpesviruses, and contribute to tumor formation. Our results have implications for understanding diseases in aquatic organisms, eutrophication, herpesviruses, and tumor formation.

## Introduction

Combined with overexploitation, habitat loss, and climate change, emerging diseases pose major impacts to biodiversity worldwide [1], [2]. Marine turtles suffer numerous population threats [3] with green sea turtles (*Chelonia mydas*) afflicted by fibropapillomatosis (FP) a debilitating tumor-forming disease [4]. While surveys show key green turtle populations are steadily growing [5], [6], FP remains widespread and its origins are unknown. Here we present a spatial epidemiology from 28 years of disease records from the Hawaiian population of green turtles. We construct time series of disease rates, address the spatial scale of variability, and examine the role of land use and invasive macroalgae.

Early hypotheses of causal factors of the disease examined vascular trematodes and toxins but results were inconclusive [7], [8]. A viral origin for FP became apparent after experiments successfully transmitted the disease using cell-free tumor extracts [9]. Later studies identified *α*-herpesviruses as the leading candidate after their DNA fragments were discovered in turtle tumors, but were absent in tumor-free turtles [10], [11]. Subsequent results also showed sampled herpesviruses had low genetic variability [11], [12] implying contact transmission, perhaps via ectoparasites [13].

Further advances to understanding this disease have been limited by the inherent complexities of epidemics and their ecosystems [14]. Infectious diseases involve individual susceptibility, exposure, infection, and immune response. These phases often operate independently; interact in nonlinear ways; and vary demographically, geographically and through time. Mass-action models [15], for example, can predict the course of many diseases by their host population density. These models are intuitive, as communicable diseases often spread rapidly in dense populations. Understanding the variability of FP, however, is likely more complicated than transmission dynamics alone. In Hawaiian green turtles, for example, FP became prevalent in the 1980s, and apparently peaked in the 1990s [16], [17] though the turtle population has grown continually [5]. Furthermore, recent phylogenetic analyses of the implicated herpesviruses show low mutability and coevolution with their turtle hosts over millions of years [12]. Investigating factors that can promote disease, such as environmental [18] or dietary conditions [19], may therefore provide insights.

**Figure 1:**
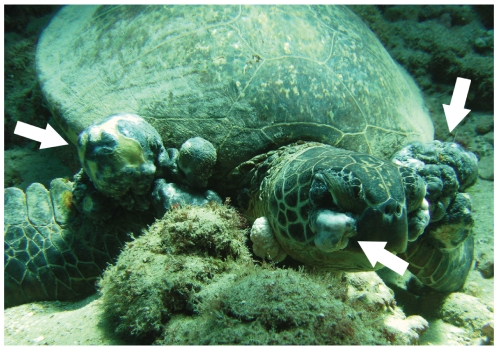
Hawaiian green turtle (*Chelonia mydas*) with fibropapillomatosis (FP) on the seafloor in Pupukea Marine Life Conservation District, North Shore, Oahu. Evidence suggests that this disease, characterized by external epithelial tumor masses (white arrows), is caused by a herpesvirus. Photo: Lacey Price/Marine Photobank, taken April 2008.

Green turtles develop FP (Fig. 1) only after recruiting to nearshore habitat [17], [20] indicating these environments are influential. Most Hawaiian green turtles hatch in the Northwestern Hawaiian Islands (NWHI, 900 km from Honolulu) and spend up to a decade in pelagic waters [21]. Juveniles recruit to nearshore waters at around 35 cm straight carapace length (SCL). Here turtles maintain spatiotemporal fidelity to specific macroalgae beds in shallow, nearshore sites [16], [22]. After reaching ∼80 cm SCL, individuals seasonally migrate to the NWHI to breed. There they spend months, afterwards return to their foraging sites in the Main Hawaiian Islands (MHI), and subsequently breed every 3–4 or more years [23]. Therefore all neritic green turtles are chronically and locally influenced by their local nearshore habitat in the MHI.

We examine FP records of green turtles stranded on Hawaiian beaches from 1982–2009 considering the uniqueness of the archipelago. Unlike stranding investigations in the southeastern USA where turtles drift considerable distances after offshore mortality [24], we assume most turtles and most population threats are proximate to coasts. The Hawaiian islands are oceanic pinnacles with no continental shelf and local fisheries bycatch is not a major threat [17]. Population and ecosystem changes are likely important considerations, however. Conservation efforts established in the 1970s preceded a dramatic population recovery, in spite of the widespread occurrence of FP [5]. Additionally during this period, invasive macroalgae bloomed across the MHI reportedly spurred by nutrient enrichment from agriculture runoff and discharged sewage [25], [26], [27]. We therefore examine the following questions. Is turtle size a risk factor for this disease? At what scales do disease rates vary in space and time? Are disease rates spatially clustered? Do epidemiological links to land use or macroalgae exist?

## Materials and Methods

### Turtle strandings data

We compiled strandings data from dead or moribund turtles reported to the National Marine Fisheries Service, Pacific Islands Fisheries Science Center [17]. These data span the entire archipelago, but we restricted the analysis to Oahu, Maui, and Hawaii due to observer coverage. We documented stranding locations from locality descriptions from 1982–1999, afterwards using global positioning system coordinates. We considered turtles FP positive when external exams identified tumors (Fig. 1) as no turtles with internal tumors lacked them externally. Demographic data were limited to size measurements. We used SCL for size and calculated it from curved carapace length (using *SCL* = 0.93*CCL, *r* ^2^ = 0.99), when only the latter was available. This yielded 3,939 records spanning 28 years containing location, disease, and turtle size data.

### Standardizing disease rates

As size is a known risk factor for FP [17], [28] we calculated the stranding frequencies of size classes through time and determined their size-specific disease rates. Understanding these relationships is essential for accurate comparisons, especially to avoid reporting differences that are merely demographic artifacts [29]. To describe changes in the strandings during the study, we grouped strandings into five equal time periods and six size classes and fit probability models to the size frequency data. We used the log-normal, gamma, and log-hyperbolic secant functions as they typify population data [30], [31]. A maximum likelihood estimator chose model parameters and an Akaike Information Criterion (AIC) ranked models [32]. To describe the relationship between size and disease rate, we retained the above time and size bins and calculated the simple disease incidence proportion in each group. We plotted disease rates against size, fit quadratic models to the data and differentiated the predicted expression to determine where rates peaked.

Next we explored the spatiotemporal variability of FP by standardizing disease rates to account for the risk factor of turtle size. Standardized disease rates for subsets of the database are local incidence proportions, corrected to the size structure of a “standard” population, we defined as the most recent decade of data. We calculated them using:
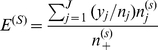
(1)where *y_j_* and *n_j_* are the FP positive and the total individuals, respectively, in each size bin (i.e., at risk) in the locally-observed population; and *n_j_* ^(*s*)^ and *n*_+_^(*s*)^ are the number of individuals at risk and the total number of individuals in the standard population, respectively [29]. Essentially, this metric weights local, size-specific disease rates according to each size class's occurrence in the standard population.

Having a comparable measure of local FP rates, *E* ^(*s*)^, we calculated their annual time series at three spatial scales: with all islands grouped, by island, and by within-island regions. Not all locations were well sampled (especially pre-1988) so we combined adjacent years with <5 records and plotted the resulting rates as the mean time. We distinguished island regions by terrestrial hydrology, identifying seven regions on Oahu (North Shore, Kahuku, Kaneohe, Waianae, South Shore, Maunalua, and Waimanalo), three on Maui (West, North, and South Maui), and two on Hawaii (Kona and Hilo). We then compared the statistical variability of the time series between spatial scales (see Table S1) ranking models using the corrected AIC (AIC_c_) [33]. This treats scale as a model factor to identify the appropriate scale for understanding disease variability.

### Characterizing land use

To understand the influence of spatial scale more acutely, we calculated disease rates in individual watersheds and examined the influence of land use. We obtained GIS coverages of land features and land use from the State of Hawaii Office of Planning [34] and the Hawaii Department of Health [35]. We combined adjacent watersheds if they shared water courses, if stranding beaches crossed boundaries, or if <5 stranding events occurred within a single area. Isolated watersheds with <5 observations were excluded. Watersheds accumulated strandings if they occurred within the boundary or <1km from shore. This provided 82 watersheds on Oahu (n = 55), Maui (n = 16), and Hawaii (n = 11).

As individual green turtles in Hawaii are repeatedly captured in the same nearshore sites [16], [22] the local ecosystem influences are likely important. We developed a nitrogen-footprint to capture the combination of factors that generate, deliver, and retain N in nearshore waters [36]. Spatially-explicit footprint statistics summarize human influences across large geographic areas [37] when other empirical records are lacking. We chose ten factors for the Nitrogen-footprint based on their known effect to nearshore ecosystems [26], [36], [38], [39]: sewage injection wells, urbanization, sugar and pineapple agriculture, intensive poultry and hog farms, cattle grazing and dairy production, aquaculture and fishponds, perennial streams and rivers, estuaries and wetlands, boat harbors, and coastal lagoons created by fringing barrier reefs. (We excluded golf courses as their major nutrient contribution is phosphorus [40] which is less important than N for ecosystem changes [36], [41] or for macroalgae [38], [42], [43].) Each watershed accumulated a nitrogen-footprint score where each contributing factor is measured, equally weighted, summed, and rescaled.

For urbanization, sugar/pineapple, cattle grazing, and poultry/hog production, the Nitrogen-footprint score is the average of the % area coverage and the % drainage coverage. We preferred this to area coverage alone as human activity tends to be clustered along coastal waters and may this may skew its impact. Perennial streams, rivers, and canals accumulate within each watershed, receiving a value of 0.5 for each contribution. We scored aquaculture/fishponds and estuaries/wetlands as the % coastline coverage of their maximum width. We scored sewage injection wells by their permitted flow rates: “major” wells are municipal facilities or wells pumping 50,000–3,000,000 gallons per day (gpd), “significant” wells pump 10,000–49,999 gpd, and “minor” wells pump 1,000–9,999 gpd. We only used wells located in “Underground Injection Control Areas,” or immediately proximate to coastal waters [35]. We scored major wells = 1, significant wells = 0.25, and minor wells = 0.025. Watersheds within an embayment or bordered by a fringing reef received a score of 1. Harbors are considered “major” if they contain >100 boat docks or accommodate large ocean going vessels (military ships, commercial cruise liners, container ships), and “minor” if not: major harbors = 1, minor harbors = 0.1.

### Geographically weighted regression models

We calculated standardized disease rates for watersheds with (1) and tested for spatial autocorrelation with Moran's Index. We built geographically weighted regression (GWR) models to compare the variable relationships within watersheds, considering that parameters themselves are influenced by surrounding areas [29], [44]. The GWR models compared disease rates in each watershed to Nitrogen-footprint values, locating parameters with a Monte Carlo search using both fixed and adaptive bandwidths [44]. Because the highest-ranked time series model grouped observations at island regions we capped neighbor influences to 10 km distance and to <15 watersheds. We ran GWR models in ArcGIS [45] and ranked models using AIC_c_.

We then examined the spatial structure of the highest-ranked model's residuals, testing for autocorrelation and potential differences between islands or from macroalgae distribution. We described macroalgal history from the known occurrence of three nonnative invasives that comprise the majority of Hawaiian green turtle diets [46], [47], [48]: *Hypnea musciformis*, *Gracilaria salicornia*, and *Acanthophora spicifera*. We documented occurrence using the definitive authority on Hawaiian rhodophytes [25] and field surveys [27]. We considered occurrence “major” if it chronically exceeded >1 km of coastline and “minor” if it did not (Celia M. Smith, personal communication). If we lacked records of these species at a location, we considered them absent. We used Moran's Index to examine residual autocorrelation and we plotted the predicted *E* ^(*s*)^ values from the GWR, coding them for island and macroalgal distribution.

## Results

### Establishing risk factors

**Figure 2:**
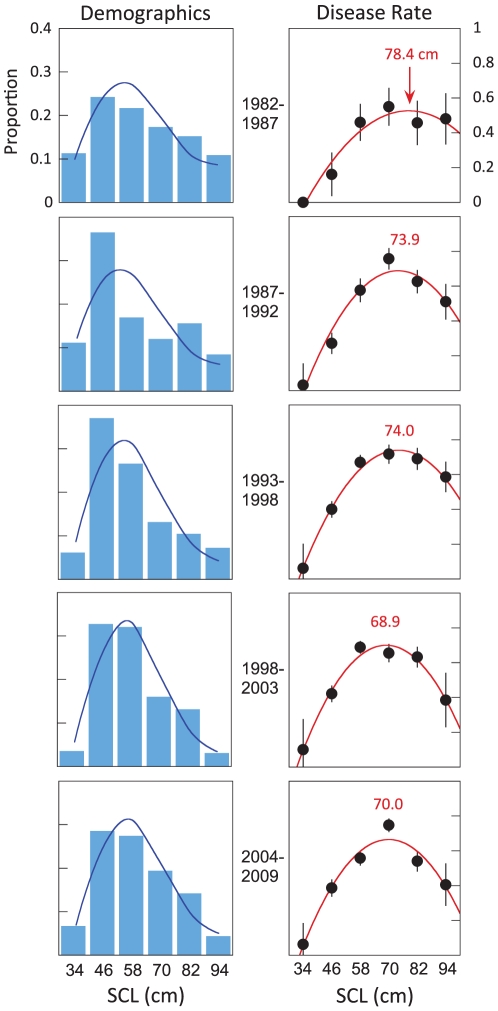
Turtle size is a consistent risk factor through time. The left panel series plots demographic data of the population during five equal time periods in six size classes (<40, 40–51, 52–63, 64–75, 76–87, >88 cm) plotted as the mean value. The fitted log-normal distribution (blue line) shows a pulse of new individuals into the population (1987–1992) and a subsequent shift to a population with more juveniles and subadults (classes 2–4). The right panels show the raw incidence proportions of the disease through time. In four of five time periods, infection rates peak in the fourth size class, corresponding to the time each size class spends in nearshore ecosystems in the main Hawaiian Islands. Red line is the quadratic fit for all periods (bars are s. e.), listed number is the size at peak rate.

Fig. 2 plots the demographic proportions of stranded green turtles through time from the islands of Oahu, Maui, and Hawaii and describes the relationship between turtle size and disease incidence. Bar plots show the demographic proportions through time fitted to a log-normal distribution, the highest-ranked model in all time steps. The second time step shows a pulse of juveniles in comparison to the previous period, and later periods show a shift towards a population skewed in favor of juveniles. This is demonstrated in that the standard deviation of the log-normal model decreases through time (see Table S2).

Simple incidence proportions of FP show disease increases with turtle size, peaks, and then declines (Fig. 2). Fitted models are first-order polynomials for all time periods, corroborating earlier results [17], [28]. All models (red lines) fit the data well (*r* ^2^ = 0.94–0.99) and as a result, all further comparisons of disease rates are standardized according to turtle size [29]. Fitted models further indicate that size at peak incidence decreases ∼10cm over the study period.

### Disease variability in space and time

**Figure 3:**
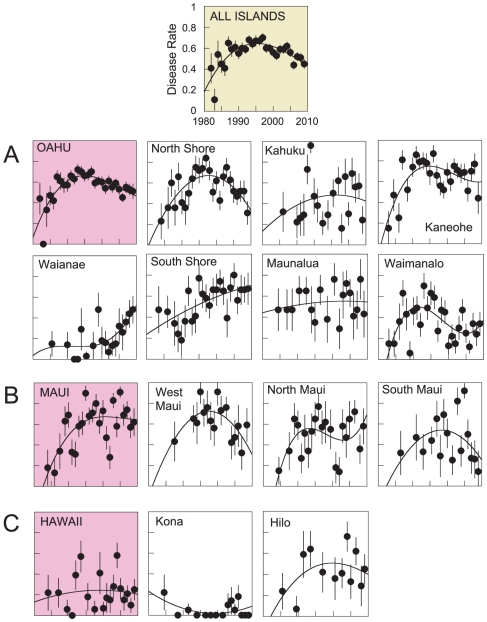
Time series of standardized disease rates show significant regional variability and suggest a local cause. All islands series (yellow plot) indicates the disease peaked - at this scale - in the mid 1990s and gradually declined thereafter. *A*, Oahu series (pink plot) is similar to the all islands trend, but regions within differ dramatically. Some Oahu regions (Waianae and South Shore) continue to increase today. Similar results are obtained for *B*, Maui and *C*, Hawaii. Trend line is the highest ranked quadratic model fit. Grouping data in space and time will likely mask important information related to the cause and impact of this disease.

Fig. 3 plots time series of standardized disease rates at varying spatial scales. Regional time series reveal dramatic local differences (Fig. 3*A–C*). The Oahu plot (Fig. 3*A*) peaks in the mid-1990s and gradually declines after, and seems to drive the signal when all islands are grouped. The Oahu trend however is quite different from regions within. North Shore, Kaneohe, and Waimanalo all peak in the 1990s and then decline; Kahuku and Maunalua gradually asymptote; and Waianae and South Shore increase. Fig. 3*B* shows a similar result for Maui where the overall Maui trend masks the recent declines of West and South Maui. The Kona region of Hawaii is nearly disease free (Fig. 3*C*). The appropriate spatial scale, therefore, seems relevant to understanding FP. Considering spatial scale as a variable, the highest-ranked model is a curvilinear fit when regions within islands are considered separately (Table S1 provides *δ*AIC_c_ values). This indicates that FP varies locally, which when considered in conjunction with spatiotemporal fidelity, encourages investigation into local causes.

**Figure 4:**
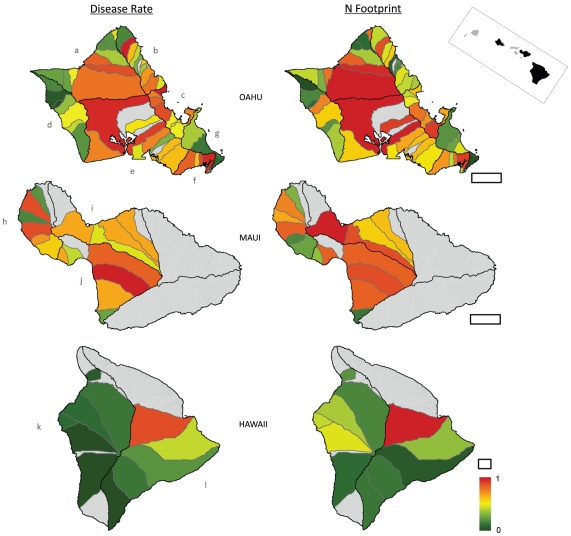
Spatial analyses reveal that disease rates are highest in watersheds where human land use impacts are greatest. Left panels plot the standardized disease rates in watersheds on all islands. Right panels display the Nitrogen-footprint index, or the combined influence of local factors that generate, deliver and retain N in coastal waters (see Methods). Geographically weighted regression (GWR) demonstrates that disease rates in watersheds increase with local and proximate eutrophication (*r* ^2^ = 0.72). Central Oahu and Maui with widespread pineapple and sugar agriculture have high disease rates. Less impacted areas in Oahu and Hawaii have lower disease rates. Island regions are: Oahu (a) North Shore, (b) Kahuku, (c) Kaneohe, (d) Waianae, (e) South Shore, (f) Maunalua, (g) Waimanalo; Maui (h) West Maui, (i) North Maui, (j) South Maui; Hawaii (k) Kona, (l) Hilo. Grey lines are watershed boundaries, black lines are hydrographic regions used in the time series (Fig. 3), and filled grey polygons are watersheds lacking turtle data. Scale bar is 10km for each island, inset map at top right displays the main Hawaiian Islands. Both panel series use the color ramp at bottom right.

Fig. 4 maps standardized disease rates and Nitrogen-footprints for local watersheds. The left series maps elevated disease rates as warm colors, with cool colors indicating low rates. High rates are clustered in all Oahu regions (save Waianae and Waimanalo) as well all three Maui regions. Four of the five highest disease rates are in Oahu watersheds - Maleakahana, Kahuku (*E* ^(*s*)^ = 0.91); Kualoa, Kaneohe (*E* ^(*s*)^ = 0.90); Kamiloiki, Maunalua (*E* ^(*s*)^ = 0.89); and Waikele, South Shore (*E* ^(*s*)^ = 0.88) – with the highest disease rate found on Maui - Hapapa, South Maui (*E* ^(*s*)^ = 0.93). By comparison, Hawaii has relatively low disease rates - with the exception of Wailuku, *E* ^(*s*)^ = 0.77. In general, the disease rate maps in Fig. 4 correspond well to the time series in Fig. 3.

The right series in Fig. 4 maps Nitrogen-footprints with warm colors symbolizing high values and cool colors, low values. Watersheds in orange and red therefore indicate the combined presence of multiple factors that generate, deliver, and retain N in coastal waters. The watersheds of central Oahu for example contained pineapple and sugar agriculture, cattle grazing, sewage injection wells, urbanization, perennial water courses, and coastal estuaries. As a result, three of the top five Nitrogen-footprint values are in this area: Paukauila, North Shore (*N_i_* = 1.0); Waikele, South Shore (*N_i_* = 0.97); and Halawa, South Shore (*N_i_* = 0.93). Table S3 provides values for all watersheds.

Watershed disease rates are spatially clustered (Moran's *I* = 0.14, *z* = 3.4, *p*<0.01) indicating spatial statistics are required. The GWR examines how Nitrogen-footprint influences disease rates within watersheds; comparing the two map series in Fig. 4. The highest-ranked model used an adaptive bandwidth kernel featuring the influence of <15 neighbor watersheds (Table S4). The Nitrogen-footprint values therefore account for much of the spatial variation (*r* ^2^ = 0.72) in observed disease rates. Importantly, the model produces randomly arrayed residuals (Moran's *I* = −0.03, *p* = 0.65) indicating no systemic model deficiencies.

**Figure 5:**
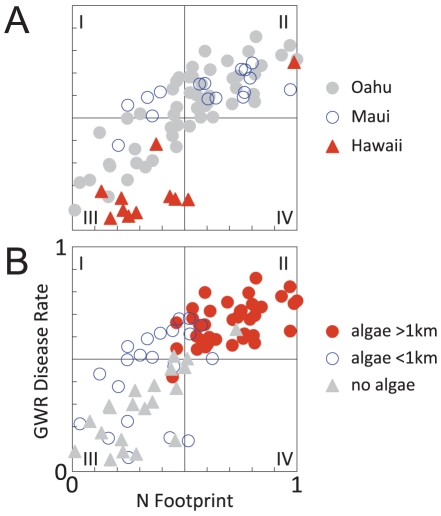
Invasive macroalgae are chronically widespread in watersheds where disease rates and Nitrogen-footprint values are elevated. *A*, GWR predicted disease rates and Nitrogen-footprints grouped by island. Oahu points are clustered (87%, 48/55) in quadrants II and III, on Maui points are clustered in quadrants I and II (94%, 15/16), and Hawaii points are clustered in quadrant III (82%, 9/11). *B*, Disease and Nitrogen-footprints are elevated where macroalgae is chronic and widespread, seen as most points are in quadrant II (88%, 35/40). Green turtles now consume nonnative macroalgae which likely sequester environmental N as arginine. Arginine is known to regulate herpesviruses and contribute to tumor formation.

Fig. 5 plots the GWR predicted disease rates for each watershed according to island and macroalgae records. Maui has the highest average disease rates with nearly 94% (15/16) of Maui watersheds clustered in quadrants I and II. Oahu watersheds are well-distributed with 87% (48/55) of points in quadrants II and III. On Hawaii, 82% (9/11) of watersheds are clustered in quadrant III. Again, Hawaii is relatively disease free with the lone triangle in quadrant II being Wailuku - the same watershed that appears reddish in both plots in Fig. 4. Fig. 5*B* shows a strong association between disease rates, Nitrogen-footprints, and macroalgae consumed by turtles. Almost 93% (37/40) of watersheds where macroalgae occurred are clustered in quadrant II where both disease rates and Nitrogen-footprint values are high. Negative correlations are also prominent. Almost 85% (17/21) of the watersheds with no such history are clustered in quadrant III. Disease rates are highest in watersheds with high Nitrogen-footprints and where nonnative algae have been chronically significant.

## Discussion

Our spatial epidemiology of FP provides four significant results: (i) turtle size is a consistent disease risk factor, (ii) disease variability is at the local scale, (iii) disease rates and land use are correlated, and (iv) the disease is linked to macroalgae. We discuss these results and potential mechanisms below.

### Variation in disease risk and rates

The observed demographic patterns of stranded turtles are likely influenced by factors besides disease. As a result, it is uncertain how these patterns relate to the population's actual population demographics. For example, the first size class never outnumbers the second (Fig. 2) which is impossible in a closed population. The pattern surely reflects the juvenile pelagic phase of the population [23] and indicates juveniles recruit to nearshore habitats in both of the first two size classes. Conservation efforts may affect stranding demographics also. The moratorium on turtle harvests since the 1970s likely contributed to the spike in juveniles in the second time step that seems to subsequently bolster larger size classes (Fig. 2).

Despite any demographic changes through the study, however, the relationship between turtle size and disease rate is consistent. The highest-ranked models show subadults are always the most affected group, but through time the size at peak disease rate decreases. This could reveal a variety of dynamics. Adults, for example, may have developed greater immunity or the disease may have become increasingly virulent, killing off younger turtles. In essence, opposite factors could produce similar patterns. The result could have little to do with epidemiology, on the other hand, and simply reflect density-dependent factors slowing somatic growth rates [22]. Future studies might examine these interactions and how risk factors themselves vary geographically.

Size-standardized disease rates reveal considerable spatiotemporal variation (Figs. 3–4) and focus attention on local disease dynamics. Though local time-series models are ranked highest, neighboring areas theoretically should be similar [29]. On Oahu - the island with the greatest coverage - the four regions on the northern half of the island have similar time series (Fig. 3*A*). The North Shore, Kahuku, Kaneohe, and Waimanalo all show peak values in the 1990s. The three southern regions of Oahu - Waianae, South Shore, and Maunalua - all peak near 2005. When disease rates are calculated by watershed, FP rates remain spatially clustered (Figs. 4, 5*A*). The Waianae, Waimanalo, and Kona regions all have low FP rates. Conversely, watersheds on Maui typically have elevated FP rates; true for several Oahu regions as well. The time series and the watershed-based analysis lead to similar conclusions: describing FP rates at large spatial scales masks important local differences.

### Limits to land use maps

The disease and Nitrogen-footprint maps have compelling similarities (Fig. 4) which the GWR test confirms. Watersheds with high disease rates tend to also have high Nitrogen-footprint values. Disease rates for Maui are relatively high across a range of Nitrogen-footprint values (Fig 5*A*). Maui is also the only island-level time series where annual disease rates surpass 90% (Fig. 3*B*). The Kona (Hawaii) and Waianae (Oahu) regions have Nitrogen-footprint values slightly above their disease rates (Fig. 4). These results may suggest variables other than those the Nitrogen-footprint accounts for factor in FP dynamics; either additional N sources or other factors entirely. Oceanographic currents, for example, could increase dilution of nutrient runoff and mitigate land use influences. However these currents are stochastic in nearshore waters and not easily characterized, especially historically. Irrigation using treated sewage might also add nutrients to ecosystems, but its use is not documented. The GWR model explains much of the variability in the data (*r* ^2^ = 0.72) and as its residuals have no spatial structure, the model does not appear to have systemic deficiencies.

The high ranking of the local time series model (Table S1) encouraged us to increase the spatial resolution to individual watersheds. This had three effects. The first is that there were not sufficient data in each watershed to calculate annual disease rates. So though we produced a fine-scale map of disease rates to individual watersheds (Fig. 4), we could not resolve the maps in time. Secondly, this naturally impacted our environmental descriptions. The Nitrogen-footprint is only a snapshot of environmental variables that vary through time. Any limitations this might impose are limited as only three of the ten components used in the Nitrogen-footprint varied considerably. These are the agricultural coverages (e.g. sugar/pineapple, cattle, poultry/hog, etc.) which actually may help explain some of the time series variability. Sugar cane and pineapple agriculture declined across the MHI during the 1990s, which broadly parallels the declines in FP rates in North Shore, North Maui, South Maui, and Hilo where these crops were formerly dominant (Fig. 3). Thirdly, the watershed maps and time-series analysis provided two sets of independent results, reinforcing their conclusions. The absence of the disease in both Kona series (Fig. 3*C*, Fig. 4) for example is also interesting. Nonnative macroalgae records on the Kona coast are few [25], [27] and land use influences there are slight (Fig. 4).

### Epidemiological Links

One explanation for our results is the dietary promotion of FP in eutrophic habitats. After 1950, native Hawaiian algae and sea grasses were displaced by nonnative species, especially in locations with elevated nutrient loads [25], [26], [27]. Nonnative macroalgae have become so dominant, that in some locations they compose >90% of green turtle diets [47], [48]. The implications of this dietary shift may be profound. When and where N is abundant, plants store excess environmental N in arginine (Arg), the only tetra-amine amino acid [49]. One study in Hawaii [50] identified two invasive algae consumed by turtles, *Hypnea musciformis* and *Ulva fasciata*, as having elevated Arg. Later isotope analysis revealed up to 43% of stored N in these species originated from discharged sewage [26]. Nonnative algae thus appear to sequester anthropogenic N, store it as Arg, and pass it on as turtle forage. This is significant as various lines of evidence implicate Arg in herpesvirus promotion and tumor growth.

Immunology and virology studies are particularly revealing. In many chronic diseases, Arg is involved in cell inflammation and immune dysfunction [51] and in promoting viral tumors [52]. But Arg is specifically important for herpesviruses which are linked to FP tumors. Experiments show that herpes does not grow without Arg [53], [54], [55], as Arg is a key building block of the viral envelope that facilitates localization, fusion, and entrance to host cell nuclei [56], [57]. Arg also seems to promote herpes-associated corneal tumors [58] and was highly concentrated in tears of rabbits with corneal herpes [59]. This is particularly relevant, as 93% of Hawaiian green turtles with FP have ocular tumors [60] (Fig. 1). How herpesviruses may promote tumor growth is uncertain, but studies show herpes may inhibit apoptosis and manipulate cell growth [61], [62]. Beyond its demonstrated role in herpesviruses, Arg is also common in a tornovirus recently found in Florida turtles with FP [63]. Histopathology studies also support an Arg-FP link. Blood assays show Hawaiian turtles with FP have elevated blood urea nitrogen compared to disease free turtles [64] which in the absence of gastrointestinal pathology [60] can indicate enhanced dietary intake of N [65]. Considered with the results of the current study, this evidence suggests nonnative macroalgae play a significant dietary role in promoting FP in marine turtles.

Fig. 5*B* clearly summarizes the links between disease rates, land use, and invasive macroalgae, yet we urge interpretative caution. Many factors contribute to the course of an infectious disease. Here we addressed the spatiotemporal variability of FP, and the environmental factors associated with promoting infections. Understanding this disease will be further advanced by examining nearshore nutrient cycling, herpesviruses, and tumor formation more acutely. Our results show that environmental factors are significant in promoting FP and suggest that eutrophic coastal ecosystems may promote herpesvirus infections among herbivores. Given the broad role of Arg in viral promotion and immune regulation our results may be significant for viral oncology more generally.

## Supporting Information

Table S1 Model results comparing temporal demographics of stranded Hawaiian green turtles, 1982–2009. Times are divided into five equal 55-month periods. N represents the strandings sample size during the period. The log-normal model is always the highest-ranked model evidence by the δAICc value is always zero. We provide log-normal parameters as a result. All models have two parameters.
(0.07 MB PDF)

Table S2 Model structure and correlates used to examine disease rate time series (Fig. 3). D is the root mean square deviation of the model from the data. N is the number of points in the analysis. The error term is assumed to be Gaussian. The highest ranking model considers disease at the regional level, within islands, and allows curvilinear variability.
(0.08 MB PDF)

Table S3 Complete data table for watersheds used in the geographically weighted regression and seen in Figs. 4–5. Data table is included as a .txt file.
(0.01 MB TXT)

Table S4 Full model results from the geographically weighted regression that allows model coefficients to vary in space. The null model is the “global” or traditional linear regression, using ordinary least squares methods. But even though this model has the lowest AICc value, it is inappropriate because the variables are spatially autocorrelated (see Results). The highest ranked model considers how a watershed's N Footprint affects disease rates within, and also factors the N Footprint of the nearest 15 watersheds. N is the number of points in the analysis, σ is the standard deviation of the model residuals.
(0.08 MB PDF)
