# Supplementary material for: Land Use, Macroalgae, and a Tumor-Forming Disease in Marine Turtles
Source: PLoS One. 2010 Sep 29;5(9):e12900. doi: 10.1371/journal.pone.0012900 (PMC2947502; doi:10.1371/journal.pone.0012900)
Supplement: Table S1 — Model results comparing temporal demographics of stranded Hawaiian green turtles, 1982–2009. Times are divided into five equal 55-month periods. N represents the strandings sample size during the period. The log-normal model is always the highest-ranked model evidence by the δAICc value is always zero. We provide log-normal parameters as a result. All models have two parameters. (0.07 MB PDF) [file pone.0012900.s001.pdf]

Factors and Covariates in model	Model Structure (deterministic part)	D	N	Parameters	δAICc
Null	$\beta_0 + \beta_1$	0.053	235	2	349
Null, polynomial (1)	$\beta_0 + \beta_1 + \beta_2$	0.051	235	3	328
Null, polynomial (2)	$\beta_0 + \beta_1 + \beta_2 + \beta_3$	0.050	235	4	328
Island	$\beta_0 + \beta_1 + \psi_i$	0.045	235	6	282
Island, polynomial (1)	$\beta_0 + \beta_1 + \beta_2 + \psi_i$	0.043	235	9	260
Island, polynomial (2)	$\beta_0 + \beta_1 + \beta_2 + \beta_3 + \psi_j$	0.042	235	12	263
Island region	$\beta_0 + \beta_1 + \omega_i$	0.026	235	24	71
Island region, polynomial (1)	$\beta_0 + \beta_1 + \beta_2 + \omega_i$	0.022	235	36	9
Island region, polynomial (2)	$\beta_0 + \beta_1 + \beta_2 + \beta_3 + \omega_j$	0.020	235	48	0

Table S1
