# Supplementary material for: Land Use, Macroalgae, and a Tumor-Forming Disease in Marine Turtles
Source: PLoS One. 2010 Sep 29;5(9):e12900. doi: 10.1371/journal.pone.0012900 (PMC2947502; doi:10.1371/journal.pone.0012900)
Supplement: Table S2 — Model structure and correlates used to examine disease rate time series (Fig. 3). D is the root mean square deviation of the model from the data. N is the number of points in the analysis. The error term is assumed to be Gaussian. The highest ranking model considers disease at the regional level, within islands, and allows curvilinear variability. (0.08 MB PDF) [file pone.0012900.s002.pdf]

Time Period	*N*	δAICc			Log-normal parameters	
		Log-normal	Gamma	Log-Sech	mean	std dev
1982-1987	232	0	472	503	6.377	0.297
1987-1992	588	0	1189	1262	6.335	0.298
1993-1998	812	0	1676	1714	6.341	0.256
1998-2003	873	0	1954	1965	6.359	0.234
2004-2009	985	0	1970	2080	6.367	0.254

Table S2
