# Supplementary material for: Land Use, Macroalgae, and a Tumor-Forming Disease in Marine Turtles
Source: PLoS One. 2010 Sep 29;5(9):e12900. doi: 10.1371/journal.pone.0012900 (PMC2947502; doi:10.1371/journal.pone.0012900)
Supplement: Table S4 — Full model results from the geographically weighted regression that allows model coefficients to vary in space. The null model is the “global” or traditional linear regression, using ordinary least squares methods. But even though this model has the lowest AICc value, it is inappropriate because the variables are spatially autocorrelated (see Results). The highest ranked model considers how a watershed's N Footprint affects disease rates within, and also factors the N Footprint of the nearest 15 watersheds. N is the number of points in the analysis, σ is the standard deviation of the model residuals. (0.08 MB PDF) [file pone.0012900.s004.pdf]

Model	Kernel Features	N	σ	r^2	δAIC_c
Null (global regression)	ordinary least squares	82	0.180	0.450	-4*
GWR, distance	fixed, 10km	82	0.150	0.701	9
GWR, distance	fixed, 5km	82	0.143	0.844	85
GWR, distance	fixed, 2km	82	0.082	0.995	1834
GWR, neighbors	adaptive, 15	82	0.155	0.723	0
GWR, neighbors	adaptive, 10	82	0.144	0.820	16
GWR, neighbors	adaptive, 5	82	0.124	0.949	233

Table S4
